# 

**Published:** 1882-07

**Authors:** 


### BOOKS AND PAMPHLETS RECEIVED.

JAHRESBERICHT DER K. CENTRAL-THIERARZNEI SCHULE IN MUNCHEN. 1880-1881. Leipzig, 1882.

A CONTRIBUTION TO THE SUBJECT OF NERVE-STRETCHING. By William J. Morton, M.D. New York. 8vo. pp. 31. Reprint from the *Journal of Nervous and Mental Diseases*, Vol. IX., No. 1. January, 1882.

CATALOGUE COLUMBIA VETERINARY COLLEGE AND SCHOOL OF COMPARATIVE MEDICINE. 1882.

THE VETERINARY JOURNAL. London.

DER ZOOLOGISCHE GARTEN. Zeitschrift für Beobachtung Pflege und Zucht der Thier Gemeinsames Organ für Deutschland und angrenzende Gebieté. Herausgegeben von der "Neuen Zoologischen Gesellschaft" in Frankfurt a M.

WOCHENSCHRIFT FUR THIERHEILKUNDE UND VIEHZUCHT. Augsburg.

SCHWEIZERISCHES ARCHIV FUR THIERHEILKUNDE UND THIERZUCHT. Bern.

REPERTORIUM DER THIERHEILKUNDE. Stuttgart.

DEUTSCHE ZEITSCHRIFT FUR THIERMEDICINE. Leipzig.

ZEITSCHRIFT FUR VERGLEICHENDE. Leipzig.

ARCHIV FUR WISSENCHAFTLICH UND PRACTISCH THIERHEILKUNDE. Berlin.

LA PRESSE MEDICALE. Paris.

THE CHICAGO MEDICAL JOURNAL AND EXAMINER. Chicago.

THE MEDICAL RECORD. New York.

THE COLLEGE AND CLINICAL RECORD. Philadelphia.

ANNALS OF ANATOMY AND SURGERY. Brooklyn.

NEW ENGLAND MEDICAL MONTHLY. Newton, Conn.

Chicago Medical Review. Chicago.
St. Louis Clinical Record. St. Louis.
Journal of Nervous and Mental Diseases. New York.
The Rocky Mountain Medical Times. Denver.
Walsh's Retrospect. Washington.
Virginia Medical Monthly. Richmond.
The Western Medical Reporter. Chicago.
The Southern Clinic. Richmond.
Nashville Journal of Medicine and Surgery. Nashville.
The Blacksmith and Wheelwright. New York.
National Live Stock Journal. Chicago.
The Breeders' Gazette. Chicago.
The Cultivator and Country Gentleman. Albany.
Indiana Farmer. Indianapolis.
Mirror and Farmer. Manchester, N. H.
Truth. Published weekly. San Francisco.
The Weekly Drovers' Journal. Chicago.

